# The Association Between Cancer Risk and Age at Onset of Smoking in Japanese

**DOI:** 10.2188/jea.JE20080093

**Published:** 2010-03-05

**Authors:** Megumi Hara, Manami Inoue, Taichi Shimazu, Seiichiro Yamamoto, Shoichiro Tsugane

**Affiliations:** 1 Department of Preventive Medicine, Faculty of Medicine, Saga University, Saga, Japan; 2 Epidemiology and Prevention Division, Research Center for Prevention and Screening, National Cancer Center, Tokyo, Japan; 3 Cancer Information Services and Surveillance Division, Center for Cancer Control and Information Services, National Cancer Center, Tokyo, Japan

**Keywords:** cancer, lung cancer, cigarette smoking, age, incidence

## Abstract

**Background:**

Young age at onset of smoking is a known risk factor for cancer; however, few studies have investigated the risk of cancer associated with onset of smoking during adolescence in Japan.

**Methods:**

We analyzed a portion of the data from a population-based cohort of 40 897 subjects aged 40 to 69 years with a history of smoking and no history of cancer at baseline.

**Results:**

During a 14-year follow-up period, 4386 total cancers and 681 lung cancers were newly diagnosed. As compared with smokers who started smoking after the age of 20 years, those who started before the age of 17 years smoked a significantly larger number of cigarettes per day for a significantly longer duration; they also had a significantly higher risk of lung cancer. The hazard ratios in men and women were 1.48 (95% confidence interval, 1.11–1.96) and 8.07 (2.34–27.85), respectively. After further adjustment for smoking amount, the associations remained significant. There was a statistically significant inverse correlation between lung cancer risk and age at onset of smoking in male current smokers whose baseline age was 50 to 59 years; no such association was detected among other age strata.

**Conclusions:**

There was no clear evidence of increased risk of cancer due to adolescent smoking. However, adolescent smoking appeared to indirectly increase lung cancer risk because it was associated with a longer duration and larger amount of smoking.

## INTRODUCTION

In Japan, the “Law Prohibiting the Smoking of Tobacco by Minors” was enacted in 1900. It prohibited the smoking of tobacco by Japanese under the age of 20 years. However, the biological importance of this age as a threshold for carcinogenesis has not been examined. Several experimental studies reported that age at smoking initiation was inversely associated with DNA adduct level^1^ and loss of heterozygosity of chromosomes^2^ in lung tissue, and that this DNA damage is associated with lung cancer risk. Although some epidemiological studies have suggested that younger age at initiation of cigarette smoking is an independent risk for lung cancer,^3^ this remains controversial. A number of studies from Japan have shown that the risk for many types of cancer rises with decreasing age at smoking onset, in comparison with nonsmokers^4^^–^^12^; however, only a few reports have examined in detail the risks associated with onset of smoking during adolescence.^5^^,^^9^^,^^11^ In addition, to our knowledge, no report has examined whether smokers who began to smoke before the age of 20 years had a higher risk of cancer than did smokers who began smoking at a later age. The objective of the present study was to examine the effect of age at initiation of smoking on cancer risk among Japanese current and former smokers.

## METHODS

### Study population

The Japan Public Health Center (JPHC)-based Prospective Study was launched in 1990 for cohort I and in 1994 for cohort II, and encompassed 5 and 6 prefectural public health center areas, respectively. The details of the study design have been described elsewhere.^13^ The study protocol was approved by the Institutional Review Board of the National Cancer Center, Japan. In the present analysis, 1 PHC area was excluded because no data on cancer incidence were available.

The study population was defined as all registered Japanese inhabitants in the 10 PHC areas, aged 40 to 59 years in cohort I and 40 to 69 years in cohort II at the beginning of each baseline survey. Initially, 133 323 subjects were identified as eligible for the study. During the follow-up period, 359 subjects were found to be ineligible for the study and excluded because of non-Japanese nationality (*n* = 51), movement out of the study area before the start of the follow-up period (*n* = 298), incorrect birth date (*n* = 6), and duplicate registration (*n* = 4), which left a final cohort of 132 964 subjects.

### Baseline survey

A baseline self-administered questionnaire survey on various health habits was conducted in 1990 for cohort I and in 1993–1994 for cohort II. A total of 106 206 participants responded to the questionnaire, yielding a response rate of 80%. A cohort of 1579 participants with incomplete information on smoking status, 62 924 never-smokers, and 806 participants with a past or present history of cancer at any site were excluded. Ultimately, 40 897 (36 673 men and 4224 women) people—28 816 current smokers (25 390 men and 3426 women) and 12 081 former smokers (11 283 men and 798 women)—remained for analysis.

The questionnaire requested information on smoking habits, including current and former smoking status, age at onset of smoking, and average number of cigarettes smoked per day. For the analysis of smoking habits, smokers were categorized as former or current smokers and by age at smoking onset (≥20, 18–19, ≤17 years).

### Follow-up

The study participants were followed until 31 December 2004. Participants who died or moved to other municipalities were identified annually through residential registers in the respective PHC areas. The cause of death was confirmed using mortality data from the Ministry of Health, Labour and Welfare.

The incidences of total cancer and lung cancer were identified by using voluntary reports from major local hospitals in the study areas and by data linkage with population-based cancer registries, with permission. Death certificate information was used as a supplementary information source. In the cancer registry system, the proportion of cases for which information was obtained only from death certificates was 4.3% during the study period. This was considered satisfactory for the present study. The site of origin and histological type were coded using the International Classification of Diseases for Oncology, Third Edition.^14^ For multiple primary cancers at different times, the earliest diagnosis was used. Using this procedure, 4386 newly diagnosed cancer cases at any site (4122 men and 264 women) and 681 newly diagnosed lung cancer cases (644 men and 37 women) were identified up to 31 December 2004.

### Statistical analysis

The descriptive statistics are presented as numbers, percentages, and means with standard deviation (SD). Pearson’s chi-square test and analysis of variance were used to evaluate the significance of differences. Dunnett’s *t*-test was used to evaluate the significance of differences between the subjects who started smoking after the age of 20 years and others.

The person-years of follow-up were counted from the date of completion of the questionnaire until the date of diagnosis of cancer at any site, lung cancer, date of death, movement out of the study area, or 31 December 2004, whichever occurred first. Cox proportional hazards regression analysis was used to calculate hazard ratios (HRs) and 95% confidence intervals (95% CI) for cancer at any site and lung cancer incidence, by age of smoking onset, and to adjust for potentially confounding variables, ie, age at baseline (continuous), study area (9 PHC areas), weekly ethanol intake (none, occasional, <150 g, 150–299 g, 300–449 g, ≥450 g for men, and none, occasional, <100 g, ≥100 g for women), body mass index (≤18.9, 19.0–20.9, 21.0–22.9, 23.0–24.9, 25.0–26.9, 27.0–29.9, ≥30.0), and green vegetable intake (every day, not every day). These variables are either known or suspected risk factors for cancer or had been found to be associated with cancer in previous studies.^10^^,^^15^^,^^16^ In addition to the above variables, a second model (HR2) also adjusted for the number of cigarettes per day (continuous). To evaluate linear trends, scored variables were included in the model. While age at smoking onset, baseline age, and duration of smoking may all have independent biological effects, it is difficult to include all 3 terms in a mathematical model because the presence of any 2 might fix the value for the third. To examine the effect of age at smoking onset on lung cancer, we first calculated the crude incidence rate of lung cancer by dividing the number of lung cancer cases by the number of person-years according to age at smoking onset (≤17, 18–19, 20, 21–22, ≥23 years) and age at baseline (40–49, 50–59, 60–69 years) in male current smokers. Next, the effect of such interaction was checked by calculating an interaction term, multiplying a dummy variable for the age at smoking onset (younger than 20 years = 1, older than 20 years = 0) by 1 variable for smoking duration (30 years or longer = 1, shorter than 30 years = 0). Finally, the cumulative lung cancer incidence curve for current male smokers, assuming different baseline hazards over the age at smoking onset, was estimated.^17^ The effect of age at smoking onset on lung cancer risk was examined by comparing the estimated curves for those who started smoking at ≤17, 18–19, 20, 21–22, ≥23 years with the same smoking duration and baseline age. The SAS statistical software program, version 9.1 (SAS Institute Inc, Cary, NC),^18^ was used to perform the statistical analyses.

## RESULTS

The baseline characteristics of the entire cohort with respect to smoking status have been reported in detail elsewhere.^12^ Briefly, in men and women, current smokers tended to consume more ethanol and have a lower body mass index than former smokers and never-smokers. In the present study, only data on current and former smokers in the entire cohort were used. Table 1 shows the baseline characteristics of the subjects according to age at smoking onset. Most started smoking after the age of 20 years, 21.6% of men and 6.4% of women started smoking between the ages of 18 and 19 years, and 6.5% of men and 1.8% of women started smoking before the age of 17 years. In men and women, participants who started smoking before the age of 20 years had smoked significantly more cigarettes, smoked longer, consumed more ethanol, had a lower body mass index, and consumed less green vegetables than those who started smoking later. In contrast, the prevalence of current smokers was highest among subjects who started smoking after the age of 20 years.

**Table 1.** Baseline characteristics of current and former smokers, by age at onset of smoking

Variable		Age at onset of smoking (36 673 men)				Age at onset of smoking (4224 women)			
	
		≥20 years (*n* = 26 387)	18–19 years (*n* = 7920)	≤17 years (*n* = 2366)	*P* value^a^	≥20 years (*n* = 3878)	18–19 years (*n* = 270)	≤17 years (*n* = 76)	*P* value^a^
No. of current smokers (%)		18 496 (70.1)	5317 (67.1)	1577 (66.7)	<0.0001	3172 (81.8)	196 (72.6)	58 (76.3)	<0.0005
No. of former smokers (%)		7891 (29.9)	2603 (32.9)	789 (33.3)		706 (18.2)	74 (27.4)	18 (23.7)	

Age, years (SD)		52.1 (8.1)	49.8 (7.8)^b^	50.8 (7.9)^b^	<0.0001	50.2 (7.9)	45.7 (6.1)^b^	47.9 (76)^b^	<0.0001

Follow-up, years (SD)		11.2 (3.8)	11.0 (3.7)^b^	11.1 (3.7)	<0.0001	11.0 (3.8)	10.9 (3.7)	10.1 (3.7)	<0.0001

No. of cigarettes per day (SD)		22.4 (11.6)	25.0 (12.2)^b^	26.3 (12.8)^b^	<0.0001	13.8 (8.5)	18.6 (10.6)^b^	20.9 (10.3)^b^	<0.0001

Duration of smoking, years (SD)		27.0 (9.8)	27.8 (9.7)^b^	30.8 (10.5)^b^	<0.0001	19.2 (9.7)	23.7 (9.5)^b^	28.4 (13.0)^b^	<0.0001

Pack-years of smoking (SD)		612.9	707.4^b^	829.4^b^	<0.0001	283.6	447.2^b^	555.5^b^	<0.0001
		(387.0)	(421.0)	(486.2)		(245.0)	(339.9)	(342.4)	

Alcohol consumption (%)									
Men	Women								
None	None	21.8	20.5	23.9	<0.0001	53.4	36.2	43.1	<0.0001
monthly	monthly	8.4	7.3	6.7		13.3	17.7	8.3	
<150 g/week	<100 g/week	21.6	19.1	17.0		15.0	14.6	5.6	
150–299 g/week	≥100 g/week	22.1	21.6	17.4		18.2	31.5	43.1	
300–449 g/week		14.0	16.6	15.2					
≥450 g/week		12.0	14.9	19.8					

Body mass	≤18.9	5.4	5.0	5.9	0.1075	10.5	14.4	9.2	0.6456
index (%)	19.0–20.9	15.7	15.7	15.3		19.4	22.2	22.4	
	21.0–22.9	26.8	25.8	26.1		25.1	24.4	30.3	
	23.0–24.9	26.8	27.3	26.0		18.6	16.7	14.5	
	25.0–26.9	15.8	16.0	15.1		13.1	11.1	11.8	
	27.0–29.9	7.7	8.3	9.2		9.1	7.8	6.6	
	≥30.0	1.9	1.9	2.4		4.2	3.3	5.3	

Green vegetable	Not every day	76.8	81.7	80.3	<0.0001	75.9	83.3	88.2	0.0012
intake (%)	every day	23.2	18.3	19.7		24.1	16.7	11.8	

SD: standard deviation.

^a^Pearson’s chi-square test and analysis of variances were used to evaluate differences between groups.

^b^Significant difference from subjects who started smoking after the age of 20 years (*P* < 0.05 on Dunnett’s *t*-test).

Table 2 shows the person-years of follow-up and HRs and their 95% CIs for incidence of cancer at all sites and lung cancer with reference to age at smoking onset. In male current smokers, the HR for total cancer incidence increased with decreasing age at smoking onset, but this association was not observed in male former smokers or female former or current smokers. Male and female smokers who started smoking before the age of 17 years had a significantly higher risk of lung cancer than did those who started after the age of 20 years. The HR1s for lung cancer among male and female current and former smokers who started smoking before the age of 17 years were 1.48 (95% CI, 1.11–1.96) and 8.07 (2.34–27.85), respectively. These increased risks were still significant even after adjustment for daily smoking dose (HR2). In a stratified analysis of men, increased HRs for smokers who started before the age of 17 years were observed in both current (HR1, 1.40; 95% CI, 1.10–1.94) and former (2.05; 1.16–3.65) smokers. However, these associations were attenuated after adjusting for pack-years of cigarette smoking, geographic area, weekly ethanol intake, body mass index, and green vegetable intake (baseline age and duration of smoking were not included in this mathematical model), and there was no increase in the HR for lung cancer in smokers who started before the age of 17 years. Regarding the association between pack-years of smoking and lung cancer risk, a significant dose–response relationship was observed (*P* < 0.0001 for the trend). As compared with smokers with a history of fewer than 20 pack-years of smoking, the HRs—after adjustment for geographic area, weekly ethanol intake, body mass index, green vegetable intake, and age at smoking onset—were 1.01 (95% CI, 0.84–1.47) for smokers with a history of 20–29 pack-years of smoking, 2.17 (1.71–2.76) for smokers with a history of 30–39 pack-years, and 3.38 (2.74–4.16) for smokers with a history of 40 or more pack-years.

**Table 2.** Hazard ratios (HR) of total cancer incidence and lung cancer incidence in men and women, by age at onset of smoking

Age at onset of smoking (years)	Person-years of follow-up	Total cancer incidence			Lung cancer incidence		
	
		No. of cases	HR1^a^ 95% CI	HR2^b^ 95% CI	No. of cases	HR1^a^ 95% CI	HR2^b^ 95% CI
Men							
Current and former smokers							
≥20	296 025.08	3049	1.00	1.00	474	1.00	1.00
18–19	87 505.42	802	1.07 (0.99–1.16)	1.06 (0.97–1.16)	114	1.02 (0.83–1.26)	1.03 (0.82–1.29)
≤17	26 189.10	271	1.08 (0.95–1.27)	1.06 (0.92–1.22)	56	1.48 (1.11–1.96)	1.40 (1.03–1.89)
		trend	*P = 0.07*	*P = 0.18*		*P = 0.03*	*P = 0.08*
Current smokers							
≥20	207 736.13	2135	1.00	1.00	389	1.00	1.00
18–19	58 719.42	542	1.12 (1.02–1.23)	1.10 (1.00–1.21)	92	1.12 (0.89–1.41)	1.06 (0.84–1.34)
≤17	17 260.66	192	1.17 (1.01–1.36)	1.14 (0.98–1.33)	41	1.40 (1.01–1.94)	1.34 (0.96–1.86)
		trend	*P = 0.005*	*P = 0.02*		*P = 0.04*	*P = 0.11*
Former smokers							
≥20	88 288.95	914	1.00	1.00	85	1.00	1.00
18–19	28 792.00	260	1.02 (0.89–1.18)	0.92 (0.74–1.16)	22	0.93 (0.57–1.53)	1.04 (0.45–2.40)
≤17	8928.44	79	0.95 (0.75–1.20)	0.76 (0.52–1.10)	15	2.05 (1.16–3.65)	2.42 (1.04–5.64)
		trend	*P = 0.89*	*P = 0.13*		*P = 0.08*	*P = 0.09*

Women							
Current and former smokers							
≥20	42 595.44	249	1.00	1.00	33	1.00	1.00
18–19	2885.40	10	0.76 (0.40–1.45)	0.61 (0.28–1.30)	1	0.75 (0.10–5.54)	0.69 (0.09–5.19)
≤17	769.31	5	1.29 (0.52–3.17)	1.44 (0.59–3.55)	3	8.07 (2.34–27.85)	8.04 (2.29–28.30)
		trend	*P = 0.90*	*P = 0.81*		*P = 0.01*	*P = 0.01*

^a^HR1 was adjusted for geographical area (categorical), weekly ethanol intake (none, monthly, <150 g, 150–299 g, 300–449 g, ≥450 g for men, and none, monthly, <100 g, ≥100 g for women), body mass index (≤18.9, 19–20.9, 21.0–22.9, 23.0–24.9, 25.0–26.9, 27.0–29.9, ≥30.0) green vegetable intake (every day, not every day), and age in years at baseline (continuous).

^b^HR2 was adjusted for all variables in HR1, plus number of cigarettes per day (continuous).

**Figure 1.**
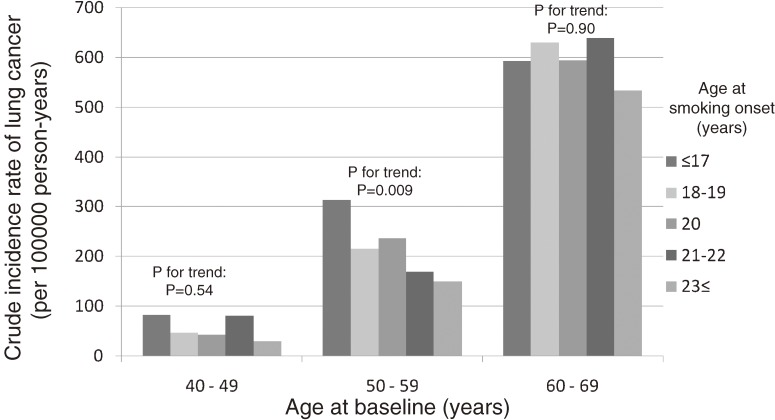
Crude incidence rate of lung cancer in male current smokers.

Figure 1 shows the crude incidence rates (per 100 000 person-years) of lung cancer by age at smoking onset and age at the baseline survey among male current smokers. The crude incidence rate of lung cancer increased with baseline age, which is believed to closely parallel smoking duration. A significant trend toward higher lung cancer risk and earlier onset of smoking was observed in male current smokers with a baseline age of 50 to 59 years, whereas no such association was found among other age strata. As compared with smokers who started smoking at the age of 20 years, the HRs of lung cancer, after adjustment for geographical area, weekly ethanol intake, body mass index, green vegetable intake, and the number of cigarettes per day in each stratum of the baseline age, were as follows. Among the group aged 40 to 49 years at baseline, the HRs were 0.69 (95% CI, 0.27–1.81) for smokers who started smoking after the age of 23 years, 1.97 (0.96–4.08) for those who started smoking at the age of 21 or 22 years, 1.06 (0.57–1.97) for those who started at the age of 18 or 19 years, and 1.63 (0.71–3.77) for those who started smoking before the age of 17 years. Among those aged 50 to 59 years at baseline, the respective HRs were 0.63 (0.44–0.91), 0.72 (0.45–1.15), 0.92 (0.64–1.30), and 1.20 (0.75–1.91). Among those aged 60 to 69 years at baseline, the respective HRs were 0.95 (0.66–1.38), 1.09 (0.71–1.66), 1.01 (0.67–1.53), and 0.99 (0.54–1.82). No significant interaction between the age at smoking onset and smoking duration was detected.

**Figure 2.**
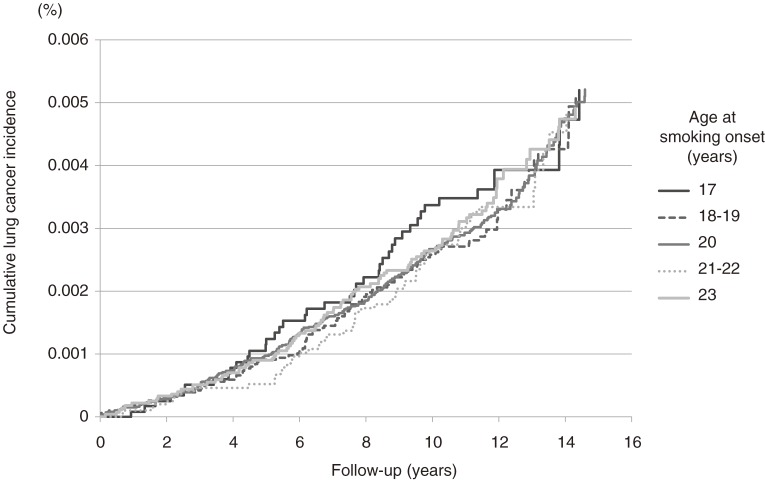
Estimated incidence of lung cancer in male current smokers.

The estimated lung cancer incidence curves are shown according to the age at smoking onset (≤17, 18–19, 20, 21–22, ≥23 years) among current male smokers whose baseline age was 40 years and smoking duration was 20 years (Figure 2). There was no clear trend toward a higher risk of lung cancer with younger age at smoking onset, but the cumulative incidence rate appeared to be higher for those who started smoking before the age of 17 years.

## DISCUSSION

Tobacco smoking is the most well-established risk factor for a number of cancers, and substantial epidemiological studies have extensively examined the risk and shown that it is correlated with smoking intensity (eg, number of cigarettes smoked per day), smoking duration, and cumulative dose (eg, pack-years).^19^^–^^22^ Although some epidemiological studies have suggested that age at smoking onset is an independent risk factor for lung cancer,^3^ this relationship remains controversial.

The present prospective cohort study found that the risk of lung cancer among smokers who started before the age of 17 years was significantly higher than that among smokers who started after the age of 20 years. It is difficult to include the age at smoking onset, baseline age, and duration of smoking in a mathematical model, even though the confounding association with smoking duration and earlier onset might influence the risk of developing lung cancer. We attempted to clarify the biological threshold of adolescent smoking on the risk of lung cancer by calculating the crude incidence rate according to the 3 baseline age strata and by estimating the lung cancer incidence curves according to age at smoking onset among smokers with the same smoking duration and baseline age. A statistically significant trend toward higher crude incidence rates of lung cancer with younger age at smoking onset was observed among male current smokers aged 50 to 59 years at baseline, but no such associations were seen among the other baseline age strata. Increased HRs of current smokers who started smoking before the age of 17 years were observed in those aged 40 to 49 and 50 to 59 years at baseline, which suggests that adolescent smoking is associated with incidence of premature lung cancer. In addition, the estimated lung cancer incidence curves indicated that the cumulative incidence rate was higher for current smokers who started smoking before the age of 17 years, especially during the follow-up period ranging from 4 to 13 years. Although there was no clear trend toward a higher risk of lung cancer with younger age at smoking onset, the possibility of increased risk due to onset of smoking during adolescence cannot be excluded. Concerning smoking amount, the increased risk of lung cancer among smokers who had started before the age of 17 years was not attenuated, even after adjustment for smoking amount. This supports the results of previous reports, which found that the duration of smoking had a greater effect on lung cancer incidence than did the number of cigarettes smoked per day.^23^^–^^25^ In the present study, it is likely that a lack of statistical power explains why younger age at smoking onset was not associated with a significantly increased total cancer risk, as indicated by the relatively low HR. However, younger age at onset of smoking is believed to increase total cancer risk because it increases the duration of smoking.

It has been suggested that prevention approaches that delay the age of smoking onset in a population could have a substantial impact on the incidence of lung cancer by shortening the duration of smoking.^25^ Individuals who start smoking at a younger age have been reported to be more likely to become a heavy smoker and a life-long smoker.^26^ A long duration of smoking, which is more likely among those who start smoking at a young age, leads to an increased risk of lung cancer. The present study showed that the proportion of current smokers among subjects who had a history of smoking was larger in smokers who had started smoking after the age 20 years than in smokers who had started before the age of 17 years, whereas both smoking duration and the number of cigarettes smoked tended to be greater among smokers who started to smoke at a younger age. A recent study of adolescents showed that symptoms of nicotine dependence develop soon after the first puff and suggested that interventions that manage dependence may be needed immediately after the onset of smoking.^27^ Presentation of the risks, with a focus on smoking in adolescents, might be useful in preventing adolescents from taking the first puff and in promoting early cessation.

We found that the relative risk of lung cancer associated with younger age at onset of smoking was higher in women than in men. Most recent prospective cohort studies show similar incidence and mortality rates in men and women with comparable smoking histories, which suggests that there is no sex difference in susceptibility to lung cancer due to smoking.^28^^–^^30^ However, the question of whether men and women with a history of smoking differ in their susceptibility to carcinogenesis with respect to age of smoking onset remains to be sufficiently investigated. Bain et al studied the age-specific incidence of lung cancer among current smokers in 4 smoking categories defined by dose per day and age at smoking onset.^28^ They found no consistent increase in the incidence rate of lung cancer, for either sex, among current smokers who started smoking at least 20 years before, among those in the same age and dose strata. In the present study, it was difficult to make a stable comparison of lung cancer risk with respect to age at onset in female smokers because there were insufficient numbers of lung cancer cases and smokers who began smoking in adolescence.

Several limitations of this study should be mentioned. First, a misclassification of age at the onset of smoking might have occurred in some cases because the study subjects who started smoking before the age of 20 years might not have answered honestly: smoking by minors (under 20 years of age) is illegal in Japan. Second, a misclassification of exposure due to changes in smoking status might have occurred because information on smoking was obtained at only one time point. If such a misclassification occurred, it could substantially change our findings. During the follow-up period, if smokers who started smoking at a younger age have a greater likelihood of becoming heavier smokers, according to the baseline survey, then the risk of adolescent smoking may have been underestimated. However, if smokers who started to smoke at a younger age tended to quit smoking during the follow-up period, then the risk would have been overestimated Third, smokers who had already died or developed cancer before the baseline study were not included. Because smoking causes cancer after regular exposure for 30 to 40 years,^31^ some smokers who started smoking before the age of 20 years might have developed lung cancer before the age of 50 years. In addition, several experimental studies reported that age at smoking initiation was inversely associated with DNA damage,^1^^,^^2^ which might be associated with a risk for early lung cancer. However, these biases may lead to an underestimation of the true risk. Finally, the depth of inhalation, cigarette type, the effects of passive smoking and environmental factors, and genetic susceptibility to cigarette smoke may also modify risks, although these factors could not be assessed. However, we did adjust for the effects of other lifestyle variables that are known or suspected to be associated with the risk of cancer.^10^^,^^15^^,^^16^ This is a clear advantage of the present study.

In conclusion, we found no clear evidence that onset of smoking during adolescence increased the risk of lung cancer or total cancer. However, this study did demonstrate that adolescent smoking may indirectly increase the risk of lung cancer, especially premature lung cancer, because it is associated with a longer duration and larger amount of smoking. The essential aspect of preventing cancer is to dissuade people from starting to smoke at any age, and to help current smokers quit as soon as possible.

## APPENDIX

The members of the Japan Public Health Center-based prospective study group (JPHC Study principal investigator: S. Tsugane) are M. Inoue, T. Sobue, and T. Hanaoka, National Cancer Center, Tokyo; J. Ogata, S. Baba, T. Mannami, A. Okayama, and Y. Kokubo, National Cardiovascular Center, Osaka; K. Miyakawa, F. Saito, A. Koizumi, Y. Sano, I. Hashimoto, and T. Ikuta, Iwate Prefectural Ninohe Public Health Center, Iwate; Y. Miyajima, N. Suzuki, S. Nagasawa, Y. Furusugi, and N. Nagai, Akita Prefectural Yokote Public Health Center, Akita; H. Sanada, Y. Hatayama, F. Kobayashi, H. Uchino, Y. Shirai, T. Kondo, R. Sasaki, Y. Watanabe, Y. Miyagawa, and Y. Kobayashi, Nagano Prefectural Saku Public Health Center, Nagano; Y. Kishimoto, E. Takara, T. Fukuyama, M. Kinjo, M. Irei, and H. Sakiyama, Okinawa Prefectural Chubu Public Health Center, Okinawa; K. Imoto, H. Yazawa, T. Seo, A. Seiko, F. Ito, and F. Shoji, Katsushika Public Health Center, Tokyo; A. Murata, K. Minato, K. Motegi, and T. Fujieda, Ibaraki Prefectural Mito Public Health Center, Ibaraki; K. Matsui, T. Abe, M. Katagiri, and M. Suzuki, Niigata Prefectural Kashiwazaki and Nagaoka Public Health Center, Niigata; M. Doi, A. Terao, Y. Ishikawa, and T. Tagami, Kochi Prefectural Chuo-higashi Public Health Center, Kochi; H. Sueta, H. Doi, M. Urata, N. Okamoto, and F. Ide, Nagasaki Prefectural Kamigoto Public Health Center, Nagasaki; H. Sakiyama, N. Onga, H. Takaesu, and M. Uehara, Okinawa Prefectural Miyako Public Health Center, Okinawa; F. Horii, I. Asano, H. Yamaguchi, K. Aoki, S. Maruyama, M. Ichii, and M. Takano, Osaka Prefectural Suita Public Health Center, Osaka; Y. Tsubono, Tohoku University, Miyagi; K. Suzuki, Research Institute for Brain and Blood Vessels Akita, Akita; Y. Honda, K. Yamagishi, and S. Sakurai, Tsukuba University, Ibaraki; M. Kabuto, National Institute for Environmental Studies, Ibaraki; M. Yamaguchi, Y. Matsumura, S. Sasaki, and S. Watanabe, National Institute of Health and Nutrition, Tokyo; M. Akabane, Tokyo University of Agriculture, Tokyo; T. Kadowaki, Tokyo University, Tokyo; M. Noda, International Medical Center of Japan, Tokyo; Y. Kawaguchi, Tokyo Medical and Dental University, Tokyo; Y. Takashima, Kyorin University, Tokyo; K. Nakamura, Niigata University, Niigata; S. Matsushima and S. Natsukawa, Saku General Hospital, Nagano; H. Shimizu, Sakihae Institute, Gifu; H. Sugimura, Hamamatsu University, Shizuoka; S. Tominaga, Aichi Cancer Center Research Institute, Aichi; H. Iso, Osaka University, Osaka; M. Iida, W. Ajiki, and A. Ioka, Osaka Medical Center for Cancer and Cardiovascular Disease, Osaka; S. Sato, Osaka Medical Center for Health Science and Promotion, Osaka; E. Maruyama, Kobe University, Hyogo; M. Konishi, K. Okada, and I. Saito, Ehime University, Ehime; N. Yasuda, Kochi University, Kochi; and S. Kono, Kyushu University, Fukuoka.
